# Risk Factors for Severe COVID‐19 Among Children and Adolescents Enrolled in Acute Respiratory Infection Sentinel Surveillance in South Africa, 2020–2022

**DOI:** 10.1111/irv.13300

**Published:** 2024-04-26

**Authors:** Kate Bishop, Susan Meiring, Stefano Tempia, Anne von Gottberg, Nicole Wolter, Jackie Kleynhans, Fahima Moosa, Mignon du Plessis, Jocelyn Moyes, Mvuyo Makhasi, Boitumelo Chuene, Aaron M. Samuels, Halima Dawood, Gary Reubenson, Heather J. Zar, Vanessa Quan, Cheryl Cohen, Sibongile Walaza

**Affiliations:** ^1^ Division of Public Health Surveillance and Response National Institute for Communicable Diseases (NICD), a division of the National Health Laboratory Service (NHLS) Johannesburg South Africa; ^2^ Centre for Respiratory Diseases and Meningitis National Institute for Communicable Diseases (NICD), a division of the National Health Laboratory Service (NHLS) Johannesburg South Africa; ^3^ School of Public Health, Faculty of Health Sciences University of the Witwatersrand Johannesburg South Africa; ^4^ MassGenics Duluth Georgia USA; ^5^ Influenza Program Centers for Disease Control and Prevention Pretoria South Africa; ^6^ School of Pathology, Faculty of Health Sciences University of the Witwatersrand Johannesburg South Africa; ^7^ Influenza Division National Center for Immunization and Respiratory Diseases, Centers for Disease Control and Prevention Atlanta Georgia USA; ^8^ Infectious Diseases Unit, Department of Medicine Greys Hospital Pietermaritzburg KwaZulu‐Natal South Africa; ^9^ Department of Paediatrics and Child Health, Rahima Moosa Mother and Child Hospital, Faculty of Health Sciences University of the Witwatersrand Johannesburg South Africa; ^10^ Department of Paediatrics and Child Health, Red Cross War Memorial Children's Hospital, and SA‐MRC Unit on Child and Adolescent Health University of Cape Town Cape Town South Africa; ^11^ DST/NRF Vaccine Preventable Diseases Respiratory and Meningeal Pathogens Research Unit (RMPRU) Johannesburg South Africa

**Keywords:** children, COVID‐19, SARS‐CoV‐2, southern Africa, surveillance

## Abstract

**Background:**

Identifying children at risk for severe COVID‐19 disease from severe acute respiratory syndrome coronavirus 2 (SARS‐CoV‐2) may guide future mitigation interventions. Using sentinel surveillance data, we aimed to identify risk factors for SARS‐CoV‐2–associated hospitalisation among patients aged ≤ 18 years with respiratory illness.

**Methods:**

From April 2020 to March 2022, patients meeting study case definitions were enrolled at four outpatient influenza‐like illness (ILI) and five inpatient severe respiratory infection (SRI) surveillance sites and tested for SARS‐CoV‐2 infection using polymerase chain reaction (PCR). Each ILI clinic shared a catchment area with its corresponding SRI hospital. Potential risk factors for SARS‐CoV‐2–associated hospitalisation were analysed using multivariable logistic regression by comparing inpatient versus outpatient SARS‐CoV‐2 cases.

**Results:**

Of 4688 participants aged ≤ 18 years, 4556 (97%) with complete PCR and HIV data were included in the analysis. Among patients with ILI and SRI, 92/1145 (8%) and 154/3411 (5%) tested SARS‐CoV‐2 positive, respectively. Compared to outpatients, hospitalised SARS‐CoV‐2 cases were associated with age < 6 months ([adjusted odds ratio (aOR) 8.0, 95% confidence interval (CI) 2.7–24.0] versus 1–4 years); underlying medical condition other than HIV [aOR 5.8, 95% CI 2.3–14.6]; laboratory‐confirmed Omicron BA.1/BA.2 or Delta variant ([aOR 4.9, 95% CI 1.7–14.2] or [aOR 2.8, 95% CI 1.1–7.3] compared to ancestral SARS‐CoV‐2); and respiratory syncytial virus coinfection [aOR 6.2, 95% CI 1.0–38.5].

**Conclusion:**

Aligning with previous research, we identified age < 6 months or having an underlying condition as risk factors for SARS‐CoV‐2–associated SRI hospitalisation and demonstrated the potential of sentinel surveillance to monitor COVID‐19 in children.

## 1 Background

By March 2022, following four distinct epidemiological waves of COVID‐19 (ancestral strain, Beta, Delta and Omicron BA.1/BA.2 variants), 3,593,644 laboratory‐confirmed cases of COVID‐19 had been reported in South Africa with 424,394 cases and 26,176 admissions identified among children aged ≤ 18 years [1].

Although children aged ≤ 18 years appear underrepresented among COVID‐19 cases and hospital admissions, the numbers affected remain high, and little is known about risk factors for severe disease among children, particularly in South Africa. Several studies have shown children to have a lower risk of severe COVID‐19 than adults [2, 3, 4]. Identified risk factors for hospitalisation in children aged ≤ 18 years include age < 1 year and the presence of underlying conditions such as chronic lung disease, neurologic disorders, cardiovascular disease, prematurity, diabetes mellitus and obesity [1, 5, 6, 7, 8, 9, 10, 11, 12, 13]. A limitation of some of these studies is that data were not consistently available on whether SARS‐CoV‐2 was causally associated with hospitalisation or whether this was an incidental finding because of testing algorithms during this time, thus potentially confounding risk factor ascertainment. Because outcomes among adults coinfected with SARS‐CoV‐2 and HIV are associated with increased severity [14, 15, 16], and the high proportion of HIV‐infected pregnant women in South Africa [17], it is important to explore the role of HIV infection and exposure as a risk factor for severe illness among children, in addition to other previously described factors.

In this study, we analyse 2 years of data from the well‐established influenza‐like illness (ILI) and severe respiratory illness (SRI) surveillance platforms with the aim to identify risk factors for SARS‐CoV‐2‐SRI–associated hospitalisation in children aged ≤ 18 years. Furthermore, we aim to compare characteristics of SARS‐CoV‐2 positive versus negative cases and highlight the potential for sentinel respiratory surveillance programmes to monitor the epidemiology of COVID‐19 in children.

## 2 Methods

### 2.1 Sentinel Respiratory Surveillance Programmes

We conducted active, prospective, outpatient surveillance for ILI [18] at four peri‐urban and urban clinics. We also conducted active prospective hospital surveillance for SRI [19, 20] at five hospitals sharing the same catchment area as the ILI clinics. These corresponding ILI and SRI sentinel sites were only available in three provinces of South Africa at the time of this study. The study population included all individuals aged ≤ 18 years enrolled into these facilities' ILI or SRI surveillance programme from 1 April 2020 to 31 March 2022 and for whom complete polymerase chain reaction (PCR) and HIV status data were available.

### 2.2 Case Definitions

The complete ILI and SRI case definitions, as well as inclusion and exclusion criteria, are presented in Tables S1 and S2. Briefly, ILI was defined as individuals presenting to an ILI sentinel site clinic with respiratory symptoms or suspected COVID‐19. SRI was defined as individuals admitted at an SRI sentinel site hospital with a clinician‐diagnosed lower respiratory tract illness (LRTI), suspected COVID‐19, suspected neonatal sepsis or presenting with symptoms associated with pertussis. To avoid potential nosocomial acquisition, a positive case was defined as laboratory‐confirmed SARS‐CoV‐2 positive PCR result from a nasopharyngeal swab collected within 48 h of hospital admission.

### 2.3 Study Procedures

Surveillance nurses and research assistants systematically screened patients presenting for care at ILI sentinel outpatient clinics from Monday to Friday (08h00–16h00) or admitted for treatment at SRI sentinel hospitals between 17h00 on Sunday and 13h00 on Friday. Screened patients who met surveillance case definitions and were eligible for enrolment were approached for informed consent. Case investigation forms, including demographic and clinical characteristics, underlying health conditions, COVID‐19 vaccination status and in‐hospital outcome, were collected for all enrolled patients through structured interviews and by reviewing medical records. Nasopharyngeal swabs were collected from enrolled patients.

### 2.4 Laboratory Procedures

Nasopharyngeal swabs (Copan Italia, Brescia, Italy) were stored in universal transport medium (UTM) (Copan Italia, Brescia, Italy) at 4°C–8°C and transported on ice packs in a cooler box to the NICD for testing. Total nucleic acids were extracted using a MagNA Pure 96 automated extractor and the DNA/Viral NA Small Volume v2.0 extraction kit (Roche Diagnostics, Mannheim, Germany). Influenza virus, respiratory syncytial virus (RSV) and SARS‐CoV‐2 were detected using real‐time reverse transcription PCR (rRT‐PCR). Until 28 February 2021, influenza and RSV were detected using the Fast Track Diagnostics (FTD) Flu/HRSV kit (Siemens, Luxembourg) and SARS‐CoV‐2 using the TIB MOLBIOL E gene assay (Roche Diagnostics, Mannheim, Germany). Starting on 1 March 2021, SARS‐CoV‐2, influenza and RSV testing were performed using the Allplex SARS‐CoV‐2/Flu A/Flu B/RSV multiplex PCR kit (Seegene, Seoul, South Korea). SARS‐CoV‐2 positive samples were further characterised by whole genome sequencing (WGS) or Allplex Variants I and II PCR testing (Seegene, Seoul, South Korea) to determine variants of concern, except where genetic material was considered too low for variant PCR testing and WGS (cycle threshold [Ct] value > 35). HIV status was confirmed through testing done by attending clinicians as part of standard care. Pretest counselling and bedside HIV testing were performed by surveillance staff for consenting patients not tested by their attending clinician. All patients found positive for HIV were linked to care.

### 2.5 Period of Enrolment

The periods of enrolment were classified according to out‐of‐wave and in‐wave periods from April 2020 to March 2022. An out‐of‐wave period was when the weekly incidence risk of SARS‐CoV‐2 was < 30 cases and the in‐wave period ≥ 30 cases per 100,000 total population from national COVID‐19 surveillance reports [21]. In‐wave period and dominant variant by epidemiological week and date were as follows: 1st in‐wave dominated by the ancestral strain: week 24 (7 June) of 2020 to week 34 (15 August) of 2020; 2nd in‐wave dominated by Beta: week 47 (15 November) of 2020 to week 5 (30 January) of 2021; 3rd in‐wave dominated by Delta: week 19 (2 May) of 2021 to week 37 (11 September) of 2021; and 4th in‐wave dominated by Omicron BA.1/BA.2: week 48 (21 November) of 2021 to week 5 (29 January) of 2022.

### 2.6 Statistical Analysis

Proportions along with univariable and multivariable random effects logistic regression accounting for clustering by province were used to (i) describe demographic and clinical factors associated with SARS‐CoV‐2 positivity among ILI or SRI cases at the nine sites and (ii) assess factors for SARS‐CoV‐2–associated SRI hospitalisation by comparing characteristics of SARS‐CoV‐2 positive outpatient ILI cases with SARS‐CoV‐2 positive hospitalised SRI cases.

The progression of time was included as a continuous variable, measuring time in months since the start of the study period to time of enrolment to account for possible improvement in clinical treatment and changes in severity as population immunity increased and susceptibility to SARS‐CoV‐2 decreased over time. Vaccination was not approved for children under 12 years of age in South Africa for the study period; children aged 12–17 years were eligible for vaccination beginning on 20 October 2021, and those aged 18 years were eligible beginning on 20 August 2021. Binary variables included race, sex, symptom duration before seeking medical care, wave period, influenza and RSV results, COVID‐19 vaccination status and underlying condition other than HIV. ‘Underlying condition’ consisted of a range of conditions including chronic lung conditions (including asthma and previous or current episodes of tuberculosis); neurological disorders (including seizure disorders, spinal cord injury and cerebral palsy); chronic renal failure or nephrotic syndrome; chronic or congenital heart conditions (including heart failure and valvular conditions); coronary artery disease; stroke; hypertension; diabetes; liver failure; any condition resulting in immunosuppression (including malignancies, sick cell disease, splenectomy, organ transplant, immunosuppressive therapy, autoimmune disease and immunoglobulin deficiency); burns; malnutrition; obesity; prematurity; other congenital disorder; and a current pregnancy. Nominal categorical variables included age group, province, HIV status and SARS‐CoV‐2 variant. Age groups were divided into categories (< 6 months, 6–11 months, 1–4 years, 5–12 years and 13–18 years) to distinguish between young infants, older infants, toddlers and primary and secondary school‐attending ages [1]. HIV status was categorised as either ‘HIV‐infected’ with a laboratory‐confirmed positive result; ‘HIV uninfected’ with a documented negative result including HIV‐exposed uninfected (HEU) children > 1 years of age and all HIV‐unexposed uninfected (HUU) children; or ‘HEU’ in children < 1 year old who are at increased risk of severe infections, particularly pneumonia, during the first year of life compared to HUU children [22, 23]. SARS‐CoV‐2 variants were categorised as ‘ancestral strain’, ‘Beta’, ‘Delta’, ‘Omicron BA.1/BA.2’ or ‘variant not assigned’ in instances where the sequencing failed quality control. Where no variant result was available because the specimen was tested at the sentinel site and was not available for variant characterisation or there was too little genetic material in the sample (PCR cycle threshold [C_t_] value > 35), we imputed the variant based on the period (COVID‐19 in‐wave) during which the specimen was collected.

Known risk factors from previous research for increased risk of SARS‐CoV‐2–associated SRI hospitalisation (age [6], underlying conditions [1]) were chosen a priori for inclusion in the final multivariable model. Additionally, other variables with *p* values < 0.2 in the univariable analysis, a threshold supported by literature [24, 25], were assessed for inclusion in the final multivariable model. A manual backward elimination process was used to determine the final multivariable model with statistical significance considered at a *p* value < 0.05. All analyses were conducted using Stata version 17.0 [26].

## 3 Results

**FIGURE 1:**
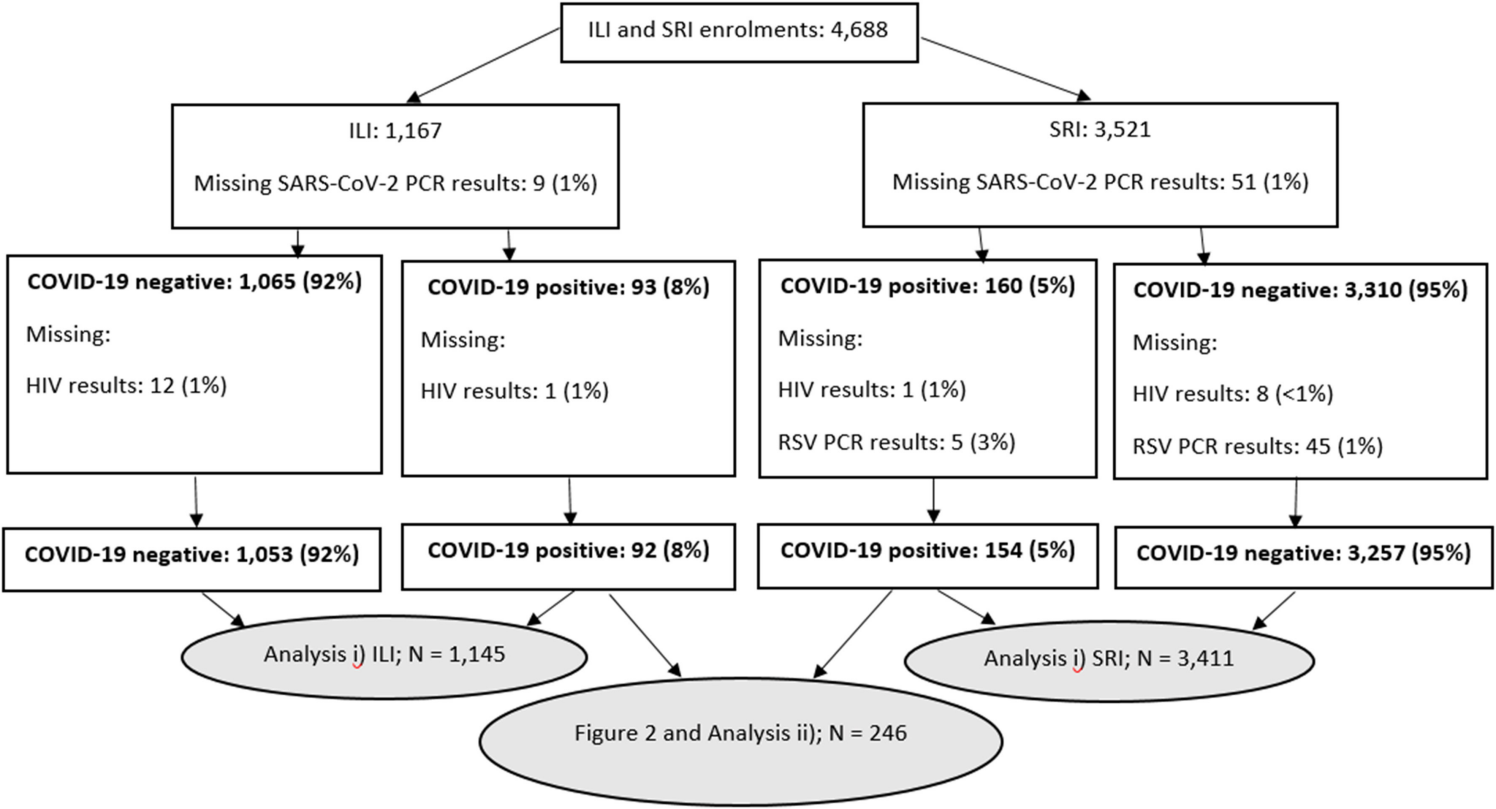
Flow chart for inclusion of participants.

From 1 April 2020 to 31 March 2022, 4688 participants aged ≤ 18 years were enrolled into participating surveillance facilities. Of the total 4688 participants, 4556 (97.2%) had complete PCR and HIV status data and were included in the analyses; 1145/4556 (25.1%) participants were enrolled from ILI and 3411/4556 (74.9%) from SRI surveillance. Cases excluded from the analysis included 110/4688 (2.3%) because of incomplete PCR data and 22/4688 (0.5%) because of incomplete data on HIV status (Figure 1).

**FIGURE 2:**
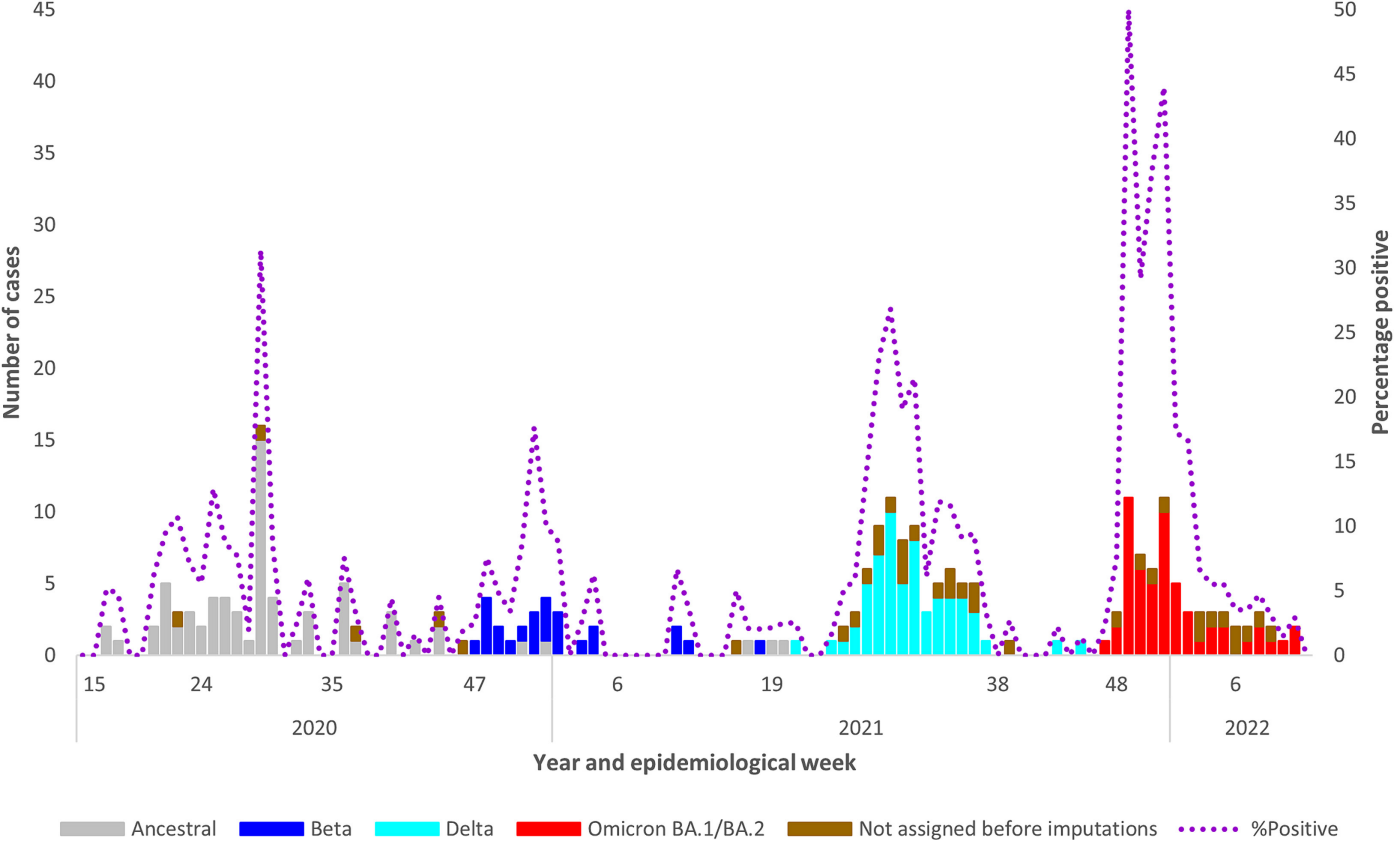
Number of laboratory‐confirmed SARS‐CoV‐2 cases in individuals aged ≤ 18 years by SARS‐CoV‐2 variant, combined data from 9 respiratory surveillance sites in 3 provinces of South Africa, 2020–2022, *N* = 246.

Among ILI and SRI participants (*n* = 4556), RSV and influenza were detected among 931 (20.4%) and 152 (3.3%) participants, respectively. Only 46/4556 (1.0%) participants were eligible to have received the COVID‐19 vaccine, and from those, 6/46 (13.0%) were vaccinated. SARS‐CoV‐2 infection was detected in 92/1145 (8.0%) ILI and 154/3411 (4.5%) SRI surveillance participants. Among all individuals testing SARS‐CoV‐2 positive (*n* = 246, 5.4%), 41 (16.7%) were during the first, 24 (9.8) during the second, 77 (31.3%) during the third, 53 (21.5%) during the fourth wave and 51 (20.7%) during the inter‐wave periods (Figure 2).

There were 85/246 (34.6%) SARS‐CoV‐2 positive cases where either the specimen was unavailable, C_t_ value was higher than the cut‐off threshold for variant characterisation, or sequencing failed. These (*n* = 85) were assigned according to the dominant variant of the wave period as follows: ancestral 24 (28.2%), Beta 1 (1.2%), Delta 9 (10.6%), Omicron BA.1/BA.2 15 (17.6%) or ‘variant not assigned’ 36 (42.4%).

On multivariable analysis, comparing SARS‐CoV‐2 negative individuals with SARS‐CoV‐2 positive cases among outpatients with ILI, the SARS‐CoV‐2 positive cases were more likely to be aged 6–11 months [adjusted odds ratio (aOR) 2.7, 95% confidence interval (CI) 1.2–6.1], 5–12 years [aOR 3.3, 95% CI 1.7–6.2] or 13–18 years [aOR 6.5, 95% CI 3.3–12.7] compared with the 1–4 years age group (Table 1). Among hospitalised patients with SRI, SARS‐CoV‐2 positive versus negative cases were more likely to be < 6 months [aOR 2.8, 95% CI 1.8–4.2], 5–12 years [aOR 2.1, 95% CI 1.0–4.1] or 13–18 years [aOR 11.3, 95% CI 4.4–28.6] compared with the 1–4 years age group. Individuals with SARS‐CoV‐2 infection were 1.5 times more likely to have an underlying medical condition compared to the SARS‐CoV‐2 negative cases [aOR 1.5, 95% CI 1.1–2.2].

**TABLE 1:** Demographic and clinical characteristics of participants ≤ 18 years with either a negative or a positive laboratory‐confirmed SARS‐CoV‐2 result presenting with ILI or hospitalised with SRI at 9 sentinel sites in South Africa, April 2020 through to March 2022 (*N* = 4556).

	Influenza‐like illness (ILI) *N* = 1145				Severe respiratory illness (SRI) *N* = 3411			
Characteristic	SARS‐CoV‐2 negative *n*/*N* (%) (*N* = 1053)	SARS‐CoV‐2 positive *n*/*N* (%) (*N* = 92)	OR [95% CI]^a^ ^,^ ^b^	aOR [95% CI]^a^	SARS‐CoV‐2 negative *n*/*N* (%) (*N* = 3257)	SARS‐CoV‐2 positive *n*/*N* (%) (*N* = 154)	OR [95% CI]^a^ ^,^ ^b^	aOR [95% CI]^a^
Age group								
< 6 months	84 (8.0)	6 (6.5)	2.2 [0.8–5.7]	2.1 [0.8–5.5]	1447 (44.4)	88 (57.1)	2.2 [1.5–3.4]	2.7 [1.8–4.2]
6–11 months	123 (11.7)	11 (12.0)	2.7 [1.2–6.1]	2.7 [1.2–6.1]	477 (14.7)	15 (9.7)	1.1 [0.6–2.1]	1.3 [0.7–2.5]
1–4 years	455 (43.2)	15 (16.3)	Reference	Reference	1128 (34.6)	31 (20.1)	Reference	Reference
5–12 years	284 (27.0)	34 (37.0)	3.7 [1.9–6.8]	3.3 [1.7–6.2]	186 (5.7)	12 (7.8)	2.3 [1.2–4.6]	2.1 [1.0–4.1]
13–18 years	107 (10.2)	26 (28.3)	7.3 [3.8–14.4]	6.5 [3.3–12.7]	19 (0.6)	8 (5.2)	14.5 [5.8–36.2]	11.3 [4.4–28.6]
Race								
Black (vs. other race)	474 (45.0)	52 (56.5)	1.6 [1.0–2.4]	—	2209 (67.8)	102 (66.2)	0.8 [0.5–1.2]	—
Sex								
Female (vs. male)	519 (49.3)	49 (53.3)	1.2 [0.8–1.8]	—	1340 (41.1)	98 (41.6)	1.0 [0.7–1.4]	—
Province								
KwaZulu‐Natal	25 (23.9)	22 (23.9)	0.6 [0.3–1.2]	—	481 (14.8)	30 (19.5)	0.8 [0.5–1.5]	—
North West	111 (10.5)	16 (17.4)	Reference	—	337 (10.4)	25 (16.2)	Reference	—
Western Cape	690 (65.5)	54 (58.7)	0.6 [0.3–1.0]	—	2439 (74.9)	99 (64.3)	0.5 [0.3–0.9]	—
Underlying medical conditions								
Underlying condition	196 (9.1)	11 (12.0)	1.4 [0.7–2.6]	1.3 [0.7–2.6]	667 (20.5)	47 (30.5)	1.7 [1.2–2.4]	1.5 [1.1–2.2]
HIV status								
HIV uninfected	1036 (98.4)	90 (97.8)	Reference	—	2881 (88.5)	131 (85.1)	Reference	—
HIV infected	9 (0.9)	2 (2.2)	3.0 [0.7–12.3]	—	69 (2.1)	5 (3.3)	1.5 [0.6–3.7]	—
HIV‐exposed uninfected	8 (2.2)	0 (0.0)	0.7 [0.1–11.8]	—	307 (9.4)	18 (11.7)	1.3 [0.8–2.3]	—
RSV and influenza								
RSV positive	121 (11.5)	2 (2.2)	0.2 [0.0–0.7]	0.3 [0.1–1.1]	794 (24.4)	14 (9.1)	0.3 [0.2–0.5]	0.3 [0.2–0.5]
Influenza positive	55 (5.2)	1 (1.1)	0.2 [0.0–1.5]	—	95 (2.9)	1 (0.7)	0.2 [0.0–1.5]	—
Duration of symptoms before presenting at healthcare facility (days)								
≥ 5 days (vs. < 5 days)	158 (15.0)	20 (21.7)	1.6 [0.9–2.6]	—	294 (9.0)	18 (11.7)	1.3 [0.8–2.1]	—

*Note:* Vaccination status is not analysed because of the low numbers eligible for vaccination.

Abbreviations: aOR: adjusted odds ratio, adjusting for age group, underlying medical condition and RSV infection; HIV‐exposed uninfected: maternal HIV infection during pregnancy in children aged < 1 year; HIV uninfected: included HIV‐exposed uninfected children aged > 1 year and all HIV‐unexposed uninfected children; ILI: influenza‐like illness; OR: odds ratio; RSV: respiratory syncytial virus included in underlying medical conditions: chronic lung conditions (including asthma and previous or current episodes of tuberculosis), neurological disorders (including seizure disorders, spinal cord injury and cerebral palsy), chronic renal failure or nephrotic syndrome, chronic or congenital heart conditions (including heart failure and valvular conditions), coronary artery disease, stroke, hypertension, diabetes, liver failure, any condition resulting in immunosuppression (including malignancies, sick cell disease, splenectomy, organ transplant, immunosuppressive therapy, autoimmune disease and immunoglobulin deficiency), burns, malnutrition, obesity, prematurity, other congenital disorder and a current pregnancy; SRI: severe respiratory infection; 95% CI: 95% confidence interval.

^a^ Random effects logistic regression used to calculate ORs.

^b^ Firth regression was used to calculate the OR where *n* = 0 in HIV‐exposed group.

On multivariable analysis, compared to SARS‐CoV‐2 outpatient cases, factors significantly associated with increased risk of SARS‐CoV‐2–associated SRI hospitalisation included age < 6 months ([aOR 8.0, 95% CI 2.7–24.0] compared with 1–4 years); being infected with the Omicron BA.1/BA.2 or Delta variants ([aOR 4.9, 95% CI 1.7–14.2] or [aOR 2.8 95% CI 1.1–7.3] compared to the ancestral strain); having an underlying medical condition [aOR 5.8, 95% CI 2.3–14.6]; or being coinfected with RSV [aOR 6.2, 95% CI 1.0–38.5] (Table 2).

**TABLE 2:** Factors included in the final multivariable model for SARS‐CoV‐2–associated SRI hospitalisation among participants ≤ 18 years of age at 4 ILI and 5 SRI sentinel sites with corresponding catchment areas in South Africa, April 2020 through to March 2022 (*N* = 246).

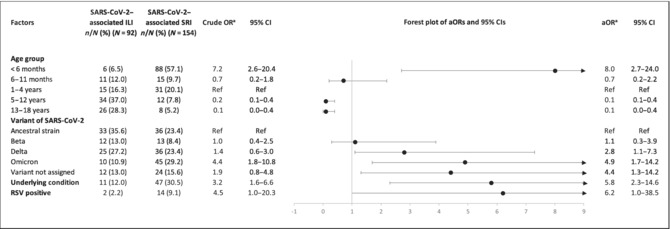

Abbreviations: aOR: adjusted odds ratio, adjusting for age group, underlying medical condition and RSV infection; ILI: influenza‐like illness; OR: odds ratio; RSV: respiratory syncytial virus included in underlying medical conditions: chronic lung conditions (including asthma and previous or current episodes of tuberculosis), neurological disorders (including seizure disorders, spinal cord injury and cerebral palsy), chronic renal failure or nephrotic syndrome, chronic or congenital heart conditions (including heart failure and valvular conditions), coronary artery disease, stroke, hypertension, diabetes, liver failure, any condition resulting in immunosuppression (including malignancies, sick cell disease, splenectomy, organ transplant, immunosuppressive therapy, autoimmune disease and immunoglobulin deficiency), burns, malnutrition, obesity, prematurity, other congenital disorder and a current pregnancy; SRI: severe respiratory infection; 95% CI: 95% confidence interval.

^a^Random effects logistic regression used to calculate ORs.

## 4 Discussion

By comparing SARS‐CoV‐2 positive individuals ≤ 18 years of age enrolled at SRI hospital surveillance sites versus those enrolled at ILI clinics, we identified risk factors for SARS‐CoV‐2–associated SRI hospitalisation, including age < 6 months, having an underlying medical condition or having the Omicron BA.1/BA.2 or Delta variants of SARS‐CoV‐2. These risk factors are similar to those associated with severe COVID‐19 disease from previous studies [1, 12, 27]. The identification of children at risk for severe COVID‐19 can inform future interventions aimed at mitigating the impact of the disease.

The distribution of SARS‐CoV‐2 positive cases from combined outpatient and hospitalised enrolments at sentinel respiratory surveillance programmes across three provinces in South Africa (Figure 2) mirrored the timing and magnitude of SARS‐CoV‐2 waves published from national data for all notified SARS‐CoV‐2 positive individuals ≤ 18 years of age [1]. This, together with the fact that we were able to identify factors for SARS‐CoV‐2–associated SRI hospitalisation, suggests that the ILI and SRI sentinel respiratory surveillance programmes in South Africa are useful for monitoring the on‐going evolution and epidemiology of SARS‐CoV‐2 in children and adolescents.

The overall prevalence of RSV coinfection with SARS‐CoV‐2 was 6.5% (16/246) and, when comparing outpatient versus inpatient sentinel surveillance data, was significantly associated with hospitalisation even after adjusting for differences in age distribution. The attributable fraction of RSV is known to be high in infants in South Africa [28], so it is possible that RSV contributed more to the cause of illness than SARS‐CoV‐2, although we cannot exclude an interaction between the two infections. Furthermore, an out‐of‐season resurgence of RSV was reported in South Africa from August to December of 2020 with continued transmission into 2021 and 2022 [29]. As described elsewhere [30, 31], influenza circulation was absent during most of the study period, likely a result of the widespread use of nonpharmaceutical interventions during this time and so we were unable to assess whether influenza and SARS‐CoV‐2 coinfection is associated with hospitalisation. Subsequently, there has been a resurgence in influenza activity with influenza transmission rising above the epidemic threshold from late April to October of 2022 [32], which will allow us to assess this association through the sentinel respiratory surveillance programme in the future.

There is minimal literature available on HIV infection or exposure as a risk factor for severe respiratory COVID‐19 among children and adolescents [33]. While the point estimate for odds of hospitalisation was higher in HIV‐infected children and HEU children compared to HIV‐uninfected children in our study, our study was underpowered to determine a significant association. Other studies have found that HEU children aged < 1 year have a greater risk of severe all‐cause pneumonia and RSV‐associated lower respiratory tract infection than HUU children [22, 23, 34, 35, 36]. Further research is needed to explore the association between paediatric HIV infection and exposure and severe COVID‐19, also considering HIV treatment and viral suppression.

A strength of the sentinel respiratory surveillance programmes is their systematic and syndromic approach to screening, assessing for eligibility and enrolling participants that are not influenced by the varying testing rates and strategies, nor by varying national recommendations for testing [1, 12, 13]. Additionally, because of the syndromic approach, asymptomatic cases, of which in childhood there is estimated to be a significant proportion compared to adults [37], would not have been eligible for enrolment, allowing us more specificity compared to laboratory‐based national all‐cause COVID‐19 surveillance in terms of SARS‐CoV‐2–associated SRI.

A limitation of our study is that it required consent from the parent or legal guardian, and so, it is possible that more severe patients, where the guardian declined consent because the child was critically ill, were excluded and so severity may be underestimated. Another limitation is that the sampling of participants is from sentinel sites in three out of nine provinces in South Africa, and so, the results may not be generalisable more broadly. Although we included 2 years of collected data, the overall number of SARS‐CoV‐2 cases was relatively low (*n* = 246), and we may have been underpowered with regards to the precision of estimated associations. Among HIV‐infected children, we were not able to take HIV viral suppression into account because of lack of available data on viral load, which may impact the association. Known risk factors were adjusted for in the analysis, except for socio‐economic status [38] which was not measured in the surveillance, and so, we expect there to be some residual confounding from this and other unknown factors, potentially resulting in incorrect estimation of associations. Additionally, in cases of RSV and influenza coinfection, we were not able to conclude whether SARS‐CoV‐2 was the infection responsible for symptoms or whether it was an incidental finding though attempts were made to rule out nosocomial acquisition.

## 5 Conclusion

In this study, the analysis of epidemiological and virological sentinel respiratory surveillance data collected between 1 April 2020 and 31 March 2022 allowed us to assess characteristics and identify risk factors for SARS‐CoV‐2–associated SRI hospitalisation in children aged ≤ 18 years seeking healthcare for respiratory illness. Further investigation is suggested to explore the association between HIV infection or an HEU status and COVID‐19 SRI. Sentinel respiratory surveillance programmes are useful in monitoring the epidemiology of COVID‐19 illness in children and adolescents.

## Author Contributions

**Kate Bishop:** Conceptualization; Data curation; Formal analysis; Project administration; Validation; Visualization; Writing – original draft. **Susan Meiring:** Conceptualization; Investigation; Supervision; Writing – review and editing. **Stefano Tempia:** Investigation; Methodology; Writing – review and editing. **Anne von Gottberg:** Investigation; Methodology; Writing – review and editing. **Nicole Wolter:** Investigation; Methodology; Writing – review and editing. **Jackie Kleynhans:** Investigation; Methodology; Writing – review and editing. **Fahima Moosa:** Investigation; Methodology; Writing – review and editing. **Mignon du Plessis:** Investigation; Methodology; Writing – review and editing. **Jocelyn Moyes:** Investigation; Methodology; Writing – review and editing. **Mvuyo Makhasi:** Data curation; Writing – review and editing. **Boitumelo Chuene:** Data curation; Validation. **Aaron M. Samuels:** Investigation; Methodology; Visualization; Writing – review and editing. **Halima Dawood:** Investigation; Methodology; Writing – review and editing. **Gary Reubenson:** Investigation; Methodology; Writing – review and editing. **Heather J. Zar:** Investigation; Methodology; Writing – review and editing. **Vanessa Quan:** Investigation; Methodology; Writing – review and editing. **Cheryl Cohen:** Conceptualization; Funding acquisition; Investigation; Methodology; Supervision; Writing – review and editing. **Sibongile Walaza:** Conceptualization; Formal analysis; Funding acquisition; Investigation; Methodology; Supervision; Writing – review and editing.

## Ethics Statement

Both the ILI and SRI protocols were approved by local human ethics research committees (HREC) according to sentinel site requirements, including the University of Witwatersrand HREC (ILI: M180832 and SRI: M140824), the University of KwaZulu‐Natal Human Biomedical Research Ethics Committee (ILI: BF 080/12 and SRI: M496/14) and the University of Cape Town Faculty of Health Science HREC (ILI: 573/2018 and SRI: 836/2014). The relevant provincial health research committees also approved the protocol. The US Centers for Disease Control and Prevention reviewed the protocols and provided a non‐research determination.

## Consent

Informed consent was obtained from parents or legal guardians in children aged < 18 years with additional assent obtained from children aged 7–17 years.

## Conflicts of Interest

H.D. reports personal fees from Merck Sharp & Dohme, Adcock Ingram, and conference attendance sponsorship from Merck Sharp & Dohme and Pfizer. H.J.Z. is supported by the South African Medical Research Council and has received funding for COVID‐related work from UK National Institute for Health GECO award (GEC111), Wellcome Centre for Infectious Disease Research in Africa (CIDRI), at the Red Cross War Memorial Children's Hospital unrelated to this body of work. J.M., C.C., N.W. and A.v.G. have received funding from Sanofi, unrelated to this body of work. N.W. and A.v.G. have received funding from the Bill & Melinda Gates Foundation (unrelated to this body of work). C.C. has received funding from PATH, South African Medical Research Council and the Bill & Melinda Gates Foundation unrelated to this work. S.W. and C.C. have received funding from the US CDC related to this work. C.C. has also received funding from the Wellcome Trust related to this body of work. K.B., S.M., S.T., J.K., M.du.P., M.M., B.C., A.M.S., G.R. and V.Q. declare no conflicts of interest.

### Peer Review

The peer review history for this article is available at https://www.webofscience.com/api/gateway/wos/peer‐review/10.1111/irv.13300.

## Supporting information


**Table S1** Influenza‐like illness (ILI) and severe respiratory illness (SRI) case definitions.
**Table S2**: Influenza‐like illness (ILI) and severe respiratory illness (SRI) inclusion and exclusion criteria.

## Data Availability

The data that support the findings of this study are available on reasonable request from the corresponding author with permission from the principle investigator, Sibongile Walaza.
