# Supplementary material for: Risk Factors for Severe COVID‐19 Among Children and Adolescents Enrolled in Acute Respiratory Infection Sentinel Surveillance in South Africa, 2020–2022
Source: Influenza Other Respir Viruses. 2024 Apr 26;18(5):e13300. doi: 10.1111/irv.13300 (PMC11046310; doi:10.1111/irv.13300)
Supplement: Supplementary file 1 — Table S1 Influenza‐like illness (ILI) and severe respiratory illness (SRI) case definitions. Table S2: Influenza‐like illness (ILI) and severe respiratory illness (SRI) inclusion and exclusion criteria. [file IRV-18-e13300-s001.docx]

Supplementary material

Table 1: Influenza-like illness (ILI) and severe respiratory illness (SRI) case definitions.

| Case Definitions | |
| --- | --- |
| ILI | SRI |
| **ILI:**  **Any age group:**  - Acute fever of ≥38 degrees Celsius and/or self-reported fever within the last 10 days AND cough; OR  - presents with acute (≤14 days) respiratory tract infection OR;  - other clinical illness compatible with COVID-19* OR;  - clinician-diagnosed suspected COVID-19:  *presents with ANY of the following respiratory symptoms: cough, sore throat, shortness of breath, anosmia (loss of sense of smell) or dysgeusia (alteration of the sense of taste), with or without other symptoms (which may include fever, weakness, myalgia, or diarrhoea)  **Pertussis:**  **Age group: < 12 months:**  - Infant with apnoea; OR  **Any age group:**  - Any patient presenting with cough of any duration AND any of the following: Paroxysms of coughing OR inspiratory whoop OR post-tussive vomiting | **SRI:**  **Age group: 2 days-<3 months**  **-** Diagnosis of sepsis/ suspected sepsis, or clinician diagnosed lower respiratory tract illness (LRTI) AND symptom any duration  **Age group: 3 months - < 5 years**  - Clinician diagnosed LRTI AND symptom onset any duration  **Age group: ≥ 5 years**  - Clinician diagnosed LRTI OR suspected COVID-19, AND symptom onset any duration  **Pertussis:**  **Age group: < 12 months:**  - Infant with apnoea; OR  **Any age group:**  - Any patient presenting with cough of any duration AND any of the following: Paroxysms of coughing OR inspiratory whoop OR post-tussive vomiting |

Table 2: Influenza-like illness (ILI) and severe respiratory illness (SRI) inclusion and exclusion criteria

| ILI |
| --- |
| **Inclusion criteria:** |
| 1. Cases (adult and paediatric) attending the clinic (Monday-Friday) and meeting the study case definitions will be eligible for inclusion 2. Patients who refuse HIV testing are still included |
| **Exclusion criteria:** |
| 1. Patients who reside outside of the catchment areas |
| SRI |
| **Inclusion criteria** |
| 1. Cases (adult and paediatric) admitted to the surveillance site and meeting the study case definitions will be eligible for inclusion 2. Sample collection must be within 48 hours of admission 3. Patients admitted from 17H00 on Sunday until 13H00 on Friday are eligible for enrolment 4. Patients who refuse HIV testing are still included |
| **Exclusion criteria:** |
| 1. Transfers directly to ICU from other hospitals and not passing through the admissions ward 2. New-borns who have not been discharged following delivery 3. Any patient who was previously enrolled in SRI less than one month ago 4. Nosocomial sepsis/ LRTI if patient was readmitted within 7 days 5. Any patient who declines 6. Out of catchment |
